# Characterization of biodegradation in a 17^th^ century easel painting and potential for a biological approach

**DOI:** 10.1371/journal.pone.0207630

**Published:** 2018-12-05

**Authors:** Elisabetta Caselli, Simonetta Pancaldi, Costanza Baldisserotto, Ferruccio Petrucci, Anna Impallaria, Lisa Volpe, Maria D’Accolti, Irene Soffritti, Maddalena Coccagna, Giovanni Sassu, Fabio Bevilacqua, Antonella Volta, Matteo Bisi, Luca Lanzoni, Sante Mazzacane

**Affiliations:** 1 Department of Medical Sciences, Section of Microbiology and Medical Genetics, University of Ferrara, Ferrara, Italy; 2 Department of Architecture and Department of Medical Sciences, CIAS Interdepartmental Research Centre, University of Ferrara, Ferrara, Italy; 3 Department of Life Sciences and Biotechnology, Section of Applied Botany, University of Ferrara, Ferrara, Italy; 4 Department of Physics and Earth Science, University of Ferrara, Ferrara, Italy; 5 National Institute for Nuclear Physics (INFN) – Ferrara Research Centre, Ferrara, Italy; 6 Musei di Arte Antica, Ferrara, Italy; 7 Restorer, Bologna and Ferrara, Italy; Universita degli Studi di Pisa, ITALY

## Abstract

It is important to characterize the microorganisms involved in biodeterioration processes to understand their effects on cultural assets and to define an efficient strategy for protecting artworks, monuments, and buildings from microbiological recolonization. In this study, we analyzed the microbial communities dwelling on the *verso* (front) and *recto* (back) sides of a 17^th^ century easel painting attributed to Carlo Bononi, an Italian artist of the first Baroque period. Cultivable bacteria and fungi colonizing the painting were isolated and identified in order to characterize the microbial community possibly involved in deteriorating the pictorial layer of the painting. The isolated bacterial strains belonged to the *Staphylococcus* and *Bacillus* genera. Furthermore, culture-dependent techniques and SEM/EDS analyses revealed the presence of filamentous fungi of the genera *Aspergillus*, *Penicillium*, *Cladosporium*, and *Alternaria*. The chemical compositions of pigments were consistent with typical 17^th^ century paintings, and some of the identified pigments, namely red lac and red and yellow earths, could be exploited as nutrient sources by painting-associated microorganisms. The study also evaluated, *in vitro*, the potential decontaminating activity of a biocompound, containing spores of *Bacillus subtilis*, *Bacillus pumilus*, and *Bacillus megaterium*. The results indicated the ability of this biocompound to counteract the growth of contaminating microorganisms that are potentially dangerous to the painting, suggesting the potential use of these microorganisms to prevent biodeterioration of artworks.

## Introduction

Biodeterioration is the alteration of organic and inorganic materials induced by the metabolic activity and growth of microorganisms [1]. Biodeterioration can play a key role in the degradation of artworks and monuments [2]. In fact, the wide variety of organic and inorganic materials contained in artworks often constitutes an optimal environment for biological colonization, which consequently causes aesthetic and structural damage [3]. Because chemo-organotrophic and/or chemo-/photo-lithotrophic microbial metabolism is dependent on the chemical nature of the materials, a complete understanding of the microbial diversity and materials in an artwork may provide useful information to design optimal restoration strategies and products [4].

Several studies have reported on the microbial colonization of damaged stone monuments [5,6], mural paintings [7–9], and frescoes [10,11], but few studies have described the microbial communities dwelling on easel paintings, namely on canvas [4,12–15] or wood panel [16,17]. Indeed, this type of artwork can possibly provide the widest range of microhabitats and nutrients suitable for the growth of microorganisms. Easel paintings are, in fact, composed of thin overlapped layers characterized by pigment grains embedded in binders that are spread on a mobile support, such as wood, canvas, or paper. The painting structure includes the pictorial layer between the protective covering varnish and the ground (or preparatory layer), which is spread on a mobile support [18,19]. Each layer is characterized by several compounds, including organic (pigment, binder, varnish) and inorganic (pigment) materials [19–21]. Consequently, microbial growth can occur in the support materials (canvas, wood, paper, etc.), materials used to create the pictorial film (pigment, oil, animal glue), or compounds used to adhere the pictorial film to the support (i.e., oil, animal glue). Bio-degradation phenomena are further favored by specific environmental conditions such as a high relative humidity that may start and/or accelerate the growth of microorganisms, which otherwise would persist in the artwork in a dormant metabolic state [4,22,23].

**Fig 1:**
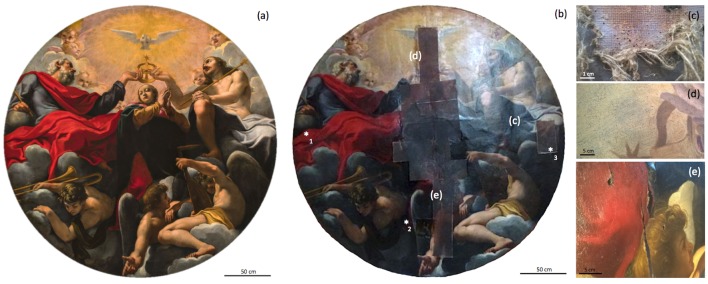
Carlo Bononi, Incoronazione della Vergine. 1616–1620, oil on canvas, 280 cm in diameter, Ferrara, Basilica of Santa Maria in Vado: a) painting (sampling points are indicated by asterisks, *); b) damage due to biodegradation (*verso*); c) craquelures network; d) lacerations, deformations, and gaps corresponding to the cloth stitching (*recto*). Details of the damage are reported on the side. Reprinted from http://www.museoinvita.it/lincoronazione-della-vergine-di-carlo-bononi/ under a CC BY license, with permission from “MuseoinVita” biannual journal, original copyright 2018.

The present study presents a case report in which we aimed to simultaneously characterize the biodeterioration phenomena and colonizing microbial community present on an ancient easel painting, “Incoronazione della Vergine,” allegedly attributed to Carlo Bononi (Ferrara, 1569?-1632) [24], an Italian artist from the Early Baroque period (Fig 1). The oil painting on canvas was completed before 1617 [25], and since then, it has remained in the Christian Basilica (15^th^ century) of Santa Maria in Vado, Ferrara, Italy. The painting is one of the “teleri” of the Basilica ceiling [26], i.e., canvas paintings of great proportions that were directly applied on the walls and painted with oil colors. This technique was typical of Venetian art and particularly used in environments where humidity might be a major factor affecting the preservation of mural paintings [27]. The artwork belongs to the original pictorial cycle planned by Bononi for the Basilica [25], and it was hung on the ceiling of the transept. Following the earthquake that occurred in Ferrara city in 2012, the painting was removed from the ceiling and left leaning against the wall of an inner niche of the church, where it continued to accumulate potential damage from biodeterioration phenomena.

In the present study, we aimed to analyze the physicochemical properties of both the pictorial materials and microbial community that resided on it and was potentially related to biodeterioration phenomena. For this purpose, we analyzed the composition of the pictorial materials and used different identification strategies to identify the microorganisms colonizing the deteriorated painting. This combined analysis provided important information about the pictorial materials and microbial colonizers, which guided the restoration project, particularly the choice of materials and appropriate biocides.

In parallel, the study also explored, by *in vitro* assays, the potential use of bacteriostatic microorganisms of the *Bacillus* genus, which are actively in hospital sanitation [28], for bioremediation purposes. This approach has been already described with good results for the treatment of historical stone works of a 20^th^ century building [29].

The results collected in our study might aid in understanding the microbial metabolic potential in the degradation process of the organic and inorganic components of paintings, providing new insights into the role of microorganisms in the deterioration of painted surfaces. Furthermore, the demonstration of the antimicrobial action of *Bacillus* bacteria against the microorganisms isolated from the painting indicates their potential for use in bioremediation. Additional studies will be needed to verify any eventual interaction of *Bacillus* bacteria with the pictorial matrix to assess the feasibility of a *Bacillus*-based treatment directly on paintings.

## Materials and methods

### Sample collection

All the samplings were performed after obtaining permission from the local Curia and authorities before starting the restoration actions. Because of the large dimensions of the artwork (280 cm in diameter) and the difficulty in moving it out of the basilica, all non-invasive analyses of the materials and the microbial samplings, as well as the restoration of the painting, were carried out *in situ*.

### Analysis of the environmental conditions

Temperature and environmental CO_2_ values were measured in close proximity to the painting using specific probes attached to an LCA-4 (ADC Co. Ltd., Hoddesdon, UK) open-system infrared gas analyzer. Photosynthetically active radiation (PAR) was measured using a portable HD 9021 Quantum Photo-Radiometer (DeltaOhm Srl, Padova, Italy) equipped with a PAR probe (400–700 nm).

### Material sampling

The sampling of pictorial materials was performed accordingly with careful visual examination of the painting surface by the restorer. The sampling procedure was performed according to the general rules of European Standard Methodology for sampling of materials from cultural heritage property [30], collecting the minimum amount required for the analysis (corresponding to 4 mm^2^) and avoiding aesthetic or structural damage to the artwork. The sampled area included the pictorial layer of one brown area and two red areas, near gaps or in a damaged but original area (Fig 1a). Three specimens were collected from the pictorial layer with a sterile surgical lancet and subsequently analyzed by both optical microscopy (OM) and scanning electron microscopy (SEM).

In order to obtain images of the stratigraphic surface of the specimens, the samples were cut with a sterile lancet without embedding them in resin to prevent the influence of laboratory resins during SEM-EDS analysis.

### Energy dispersive X-ray fluorescence (EDXRF)

Energy dispersive X-ray fluorescence (EDXRF) was used to investigate the chemical composition of the materials used by the artist. The measurements were carried out *in situ* with a Bruker Artax 200 portable energy dispersive X-ray fluorescence spectrometer (Bruker, Billerica, Massachusetts, USA) equipped with a molybdenum X-ray tube and Peltier-cooled silicon drift detector. The air-cooled X-ray tube was operated at 30 kV and 50 kV with a current of 1300 μA and 700 μA, respectively, and measuring time of 120 s. Helium gas flow was used to detect the light elements from Na (Z = 11).

### Material analysis using optical and electron microscopy

Microsamples were first studied using optical microscopy (OM) to identify the precise color nuances and the different overlapped pictorial layers. Observations were performed with a stereomicroscope (Optika SZ6745TR; Optika Srl, Bergamo, Italy) equipped with a Moticam 2005, 5.0 Megapixel Digital Color Camera (Motic, Hong Kong, China) and the Moticam Image Plus 2.0 Digital Microscopy Software Suite (Motic), at 80X total magnification.

Microsamples were also examined by SEM) using a Zeiss EVO MA 15 (Zeiss, Jena, Germany) equipped with a lanthanum hexaboride (LaB6) electron source coupled to the Oxford AZtec 3.3 EDS (Energy Dispersion Spectroscopy) analysis suite (Oxford Instruments, Abingdon, United Kingdom). To avoid interference by materials during SEM-EDS analyses, the samples were not embedded in resin. Similarly, to preserve the original surface characteristics, the specimens, mounted on stainless steel SEM stubs using carbon tape, were not coated with gold or carbon. SEM microphotographs were acquired at a 20 kV accelerating voltage with a working distance of 8.5 mm and by secondary electrons (SE) and backscattered electrons (BSE). The EDS analysis was conducted at 20 kV with a working distance of 8.5 mm, acquiring data on both spot areas and maps. SEM/EDS analyses were carried out at the Electron Microscopy Laboratory of the Department of Engineering—Department of Physics and Earth Science (University of Ferrara, Italy).

### Microbiological sampling

In order to identify and characterize the colonizing microbial communities and determine their relationships with colonized substrates, a non-invasive sampling technique was used. Considering that the growth of microorganisms on artworks depends on the environmental conditions and chemical nature of the substratum [4,12], samples were collected from different colored areas on the *recto* of the painting and from areas with noticeable signs of alteration on the *verso*.

Microbiological samples were collected from the painting by gently rubbing the sampled areas with sterile rayon swabs [31], which were then placed in dry sterile tubes and used to inoculate tryptic soy agar (TSA) and Sabouraud agar (SDA) plates. Inoculated plates were then incubated for 2–5 days at room temperature to allow the growth of collected microorganisms.

In addition, small fragments of materials from the damaged *verso* area (cotton fibers, paper) were collected and directly placed on agar plates for the isolation of microorganisms.

In parallel, microbiological samples for the isolation of autotrophic and heterotrophic microorganisms were collected by gently rubbing the sampled areas with special sterile swabs prepared under a laminar flow hood (Antares 48, Steril) for the non-invasive collection of biological material. The swabs were then used to inoculate Petri dishes containing MA [32] or Mego [33] agar media to promote the growth of autotrophic or heterotrophic microorganisms, respectively. All manipulations were carried out under the laminar flow hood described above. Plates inoculated for the isolation of possible autotrophic microorganisms were maintained at 25°C inside a climatic chamber with a 16–8 h light-dark photoperiod (30 μmol_photons_ m^-2^ s^-1^ of PAR radiation), while plates used for the isolation of heterotrophic organisms were maintained at the same temperature outside the climatic chamber. Microorganisms were checked every 7–14 days up to 75 days after the inoculation in the Petri dishes.

### Microorganism characterization

Collected microorganisms were characterized by OM, SEM, and biochemical typing. In particular, molds and yeasts were identified by OM and SEM. For SEM analyses, the biological material grown in the Petri dishes was fixed in 2.5% glutaraldehyde, prepared in 0.1 M phosphate buffer (pH 7.4), and post-fixed in 2% osmium tetroxide prepared in the same buffer. After dehydration in an ethanolic series, samples were mounted onto stabs and gold coated with an Edwards S150 sputter. Bacteria were identified by OM following Gram staining, SEM, and analysis of biochemical activities using specific biochemical tests (API, Biomerieux). OM observations were conducted with a Zeiss Oxiophot photomicroscope. SEM observations were conducted at 20 kV with a Zeiss Evo 40 scanning electron microscope at the Electron Microscopy Centre of the University of Ferrara.

### *In vitro* analysis of the antimicrobial activity of a *Bacillus*-based biocompound

The formulation of the employed bioactive product comprised spores of *Bacillus subtilis*, *Bacillus pumilus*, and *Bacillus megaterium*, with a fixed concentration of 5 x 10^7^ CFU per ml of product, and it was supplied by Copma srl (Ferrara, Italy). This optimal formulation was established in previous studies, which demonstrated that it is active as surface sanitizing agent against most types of mycetes and bacteria [28,34,35].

The concentrated product was diluted in sterile water 1:100 prior to use and finally spread on TSA plates together with the microorganisms isolated from the painting surfaces. After 2 to 5 days of incubation at room temperature, the growth of microorganisms was evaluated by plate observation and CFU enumeration.

## Results

### General state of the painting

The painting was removed from the ceiling where it was originally placed, after an earthquake that occurred in 2012, and since, then it has remained by a wall in an inner niche of the church. Temperature and CO_2_ concentration values measured in the niche in close proximity to the painting indicated that the conditions were suitable for microbial growth, as expected (Table 1).

**Table 1:** Temperature (T, C°) and carbon dioxide (CO_2_, ppm) values detected at different areas of the painting.

Location of measurement area		T (°C)	CO_2_ (ppm)
*Recto* of the painting	Upper area, left (red color)	26.75	589
	Upper area, left (yellow color)	26.75	590
	Upper area, right (yellow color)	26.80	607
	Central area, left (red color)	26.70	579
	Central area (black/dark color)	26.70	583
	Central area, right (dark color)	26.80	606
	Lower area, left (light color)	26.80	638
	Lower area, right (light color)	26.95	641
*Verso* of the painting	Upper area, left	26.90	652
	Upper are, right	26.90	656
	Central area, left	26.85	658
	Central area, right	26.90	651
	Lower area, left	26.90	647
	Lower area, right	26.90	656

The main areas of deterioration were identified on the *verso* (back) of the painting, rather than on the *recto* (front). In particular, the *verso* contained structural damage mainly due to the exposure of the canvas to a fissure in the pitched roof and the dome ceiling that allowed birds, insects, and rodents to penetrate the ceiling and damage both the original artwork and the materials used in a previous restoration operation (performed in 1995) (Fig 1b). Instead, the *recto* did not contain severe damage to the pictorial layer, except for an aging craquelure network (Fig 1c) and some small lacerations, deformations, and gaps in the pictorial film that mainly corresponded to the stitching of clothes composing the entire canvas support (Fig 1d) [36].

### Material characterization

The artwork had a color palette composed of six color shades: red, yellow, brown, blue, green, and white (Fig 1). The non-invasive nature of the EDXRF technique allowed the analysis of more than 100 points on the *recto* and *verso* of the painting, resulting in the determination of the elemental composition of the employed materials. Furthermore, to better appreciate and understand the complexity of the pictorial film, because of the overlapped painted layers, a stratigraphic analysis of two cross-sectioned samples was performed using optical microscopy and scanning electron microscopy.

**Fig 2:**
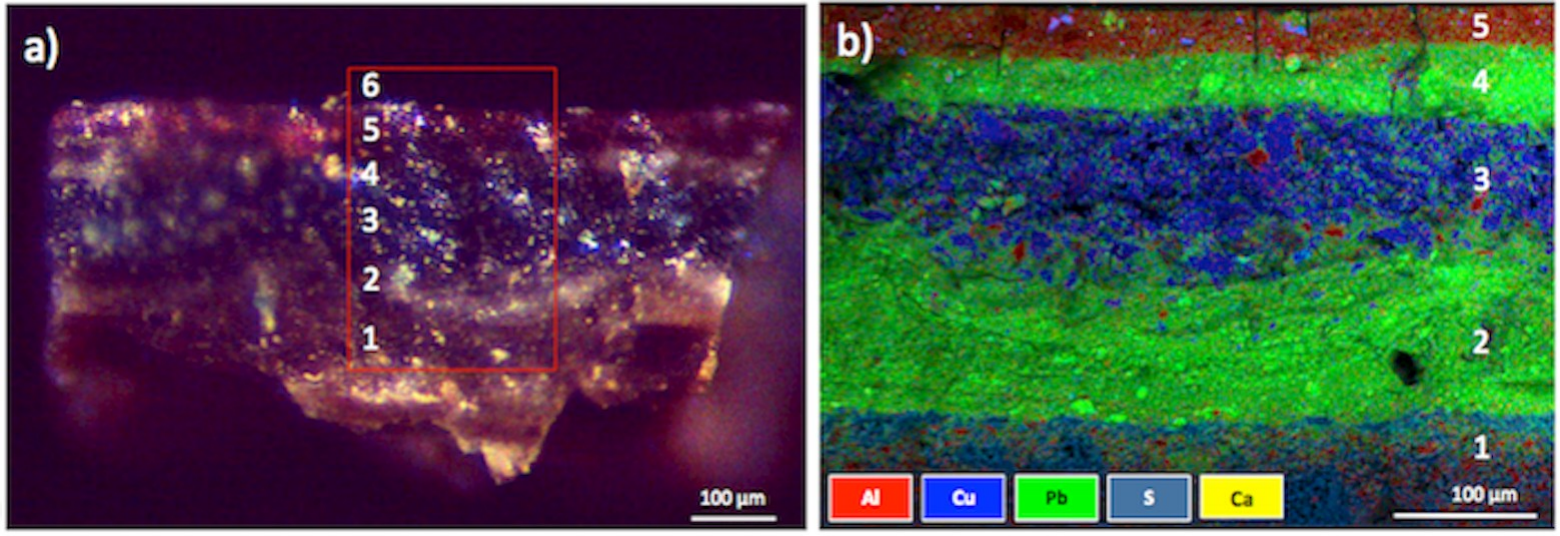
Cross-section of the sample collected from the red clothing of the Virgin, showing several overlapped pictorial layers. a) image obtained by optical microscopy (original magnification 80X), layers are indicated by the numbers 1–6, indicating the preparatory layer (1), white layer (2, 4), blue layer (3), red layer (5), and covering varnish (6); b) elemental chemical map obtained by SEM-EDS analysis. Individual chemical elements are indicated by specific colors (Al, red; Cu, blue; Pb, green, S, grey, Ca, yellow). Numbers 1–5 indicate the preparatory layer (1), white layer (2, 4), blue layer (3), and red layer (5).

The analysis confirmed that the ground preparatory layer, which was applied over the canvas support, was characterized by gypsum (CaSO_4_ ·2H_2_O) (Fig 2a and 2b layer 1), a common sulfate mineral specifically used for the ground layer of paintings [18,37–39]. The investigation also revealed that the artist used a colored ground preparation, in which gypsum was mixed with earth pigments to confer the brownish nuances (Fig 2a and 2b; layer 1). Earth pigments are a group of pigments based on a wide range of minerals, which give them their different color shades (yellow, orange, red, brown, black, and green) [38]. This pigment category includes ochres, siennas, umbers, wads, green earths, and the respective pigments obtained by roasting them [38,39]. The red/brown variety is primarily composed of clay minerals (aluminosilicates) mixed with oxides of iron [38,39]. The mineral hematite (Fe_2_O_3_) is the principal coloring component in this pigment, although it may contain other minerals such as goethite (FeO(OH)) and manganese dioxide (MnO_2_) that respectively confer a yellow-orange and dark-brown shade to the earth pigments [38,39].

Like the preparatory layer, the red and brown colors of the pictorial layer were obtained with iron oxide-rich earths. Furthermore, the strong correlation between the counting rates pertaining to Fe and Mn for the brown areas indicates the use of an umber, in which iron and manganese are always found in the same weight ratio [40]. In the yellow areas of the painting, EDXRF analysis confirmed the presence of Fe, Mn, Si, and Al, indicating the use of yellow ochre [38,39].

In addition to natural earth pigments, the analysis confirmed the presence of artificial pigments, such as lead tin yellow. Tin and lead were detected in many yellow parts of the painting, such as the crown, pastorals, trumpet, and clothing of the Angel. These elements are indicators of lead tin yellow (Pb_2_SnO_4_), also known as “giallolino” or “giallorino” (original Italian terms) and particularly employed between the 14^th^ and 18^th^ centuries [38,39].

In red areas, the analysis confirmed that the artist used cinnabar (HgS) and minium (Pb_3_O_4_) in particular to create a brilliant red color, namely in the clothing of the Creator. In fact, SEM/EDS analysis of the cross-sectioned surface of sample 1 (Fig 1a), taken from the light-red cloak of the Creator, showed that the red pictorial layer was composed of two red layers: the upper layer was characterized by cinnabar, which has been used since the Roman period [38,39]; the latter, which was applied over the preparation, was instead characterized by lead, which is typical of minium, commonly obtained by roasting a white lead-based pigment [38,39,41], such as lead white (2PbCO_3_•Pb(OH)_2_). Lead white was also detected in the painting among the white pigments, and it was mixed with other pigments to create and modify color hues; for example, lead white was mixed with red earths to obtain the pink tint of the skin of the characters.

Moreover, the analysis confirmed that the artist covered the preparation with several thin coats of paint of different shades. A chromatic change can be detected in the painting composition when the number of overlapped pictorial layers increases: the cross section of sample 2, collected from the red clothing of the Virgin, had four pictorial layers between the covering varnish and the preparation (Fig 2a). Each of these layers had a different chemical composition, suggesting the use of lead white for the white layer (Fig 2a and 2b; layers 2, 4) and a copper-based pigment for the blue layer (Fig 2a and 2b; layer 3). Copper was also detected by EDXRF in the blue and green hues of the painting, suggesting the use of copper carbonate hydroxides pigments [38,39,42,43].

In the red upper layer (Fig 2a and 2b layer 5), SEM-EDS analysis confirmed the presence of aluminum, potassium, and sulfur. The high amount of Al and the scarcity of heavy chemical elements may be indicators of red lac [37,44,45], a pigment traditionally used as glazing material [45] and employed by Bononi in other artworks placed in the Basilica [46,47]. Red lac (or red lake) is a dye-based pigment [38,48] principally derived by the precipitation of natural organic dyestuffs obtained from various insects (e.g., *Kermes vermilio*) and plants (e.g., *Rubia tinctorum*) with alum, generally potassium aluminum sulfate AlK(SO_4_)_2_•12H_2_O [44,49,50]. In the lac compounds, the alum matrix is used as a reagent to form the substrate for the dyestuff [44], whereas the principal coloring matter is composed of the laccaic acids A-F from the dyestuffs [38]. Laccaic acids A-F refer to a group of closely related anthraquinone compounds [38,51,52], and the biodegradation of anthraquinone-based dyes has been reported to be associated with some fungi and bacteria [53,54]. The same might apply also to red lac, which may be subject to biodegradation problems, such as decolorizing [54,55]. Furthermore, other detected pigments may also be subject to biodegradation, such as red and yellow ochres, as has been reported for some species of the *Staphylococcus* and *Bacillus* genera (i.e., *Staphylococcus hominis* and *Bacillus cereus*), possibly resulting in pigment fading [14].

### Microbiological analyses

Visible degradation related to microbial activity was mainly detected on the *verso* of the painting, where white and grey spots involved both the original canvas, glue paste, and canvas used for the re-lining intervention carried out in 1995 (Fig 1b). The *recto* of the painting did not show chromatic alterations of the pictorial layer.

**Fig 3:**
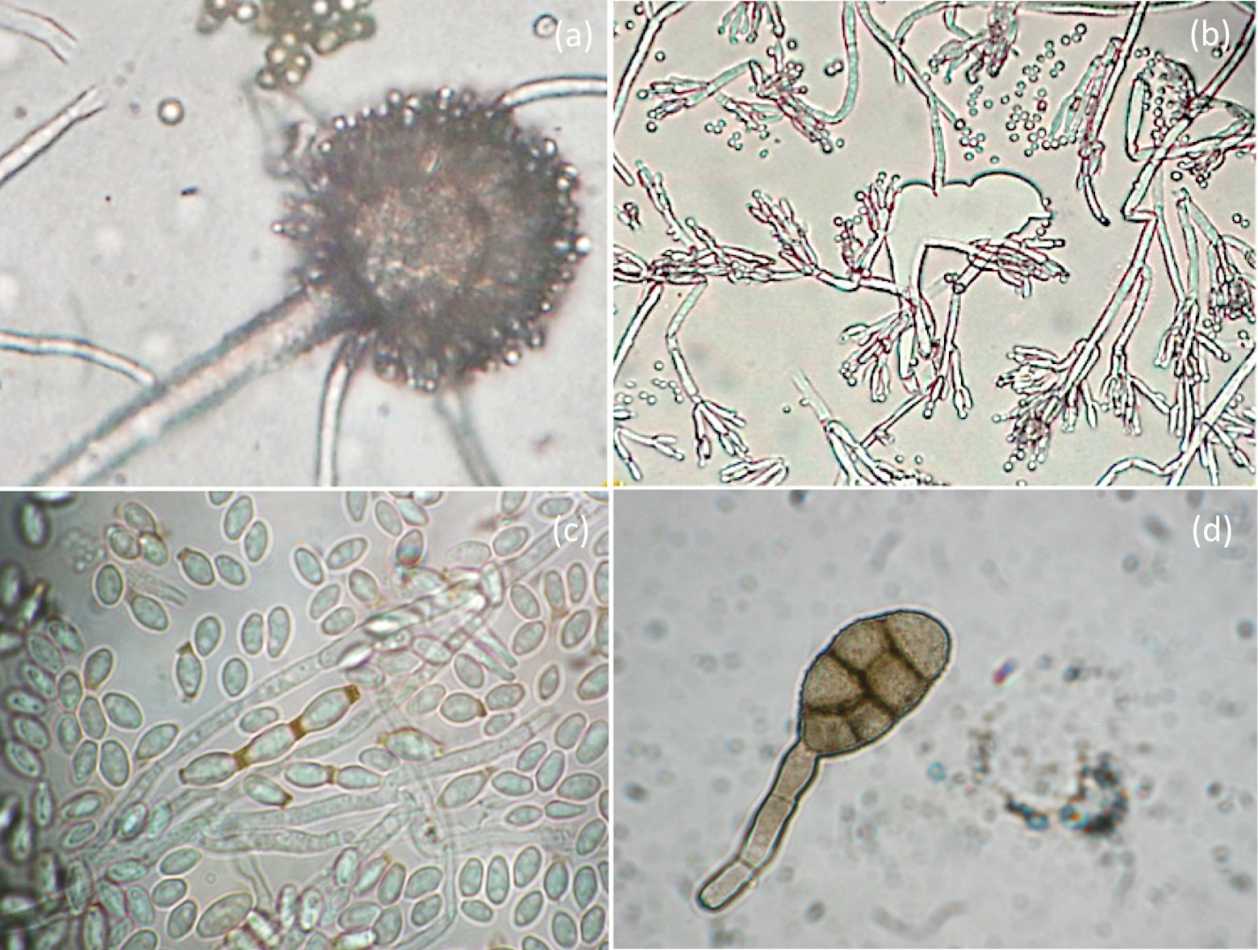
OM analysis of molds isolated from the *recto* of the painting. Photomicrographs show: a) conidial heads of *Aspergillus* spp. (original magnification 100X); b) conidial heads of *Penicillium* spp. (original magnification 100X); c) elliptical conidia ascribable to *Cladosporium* spp. (original magnification 100X); d) club-shaped and septate spores of *Alternaria* spp. (original magnification 100X).

**Fig 4:**
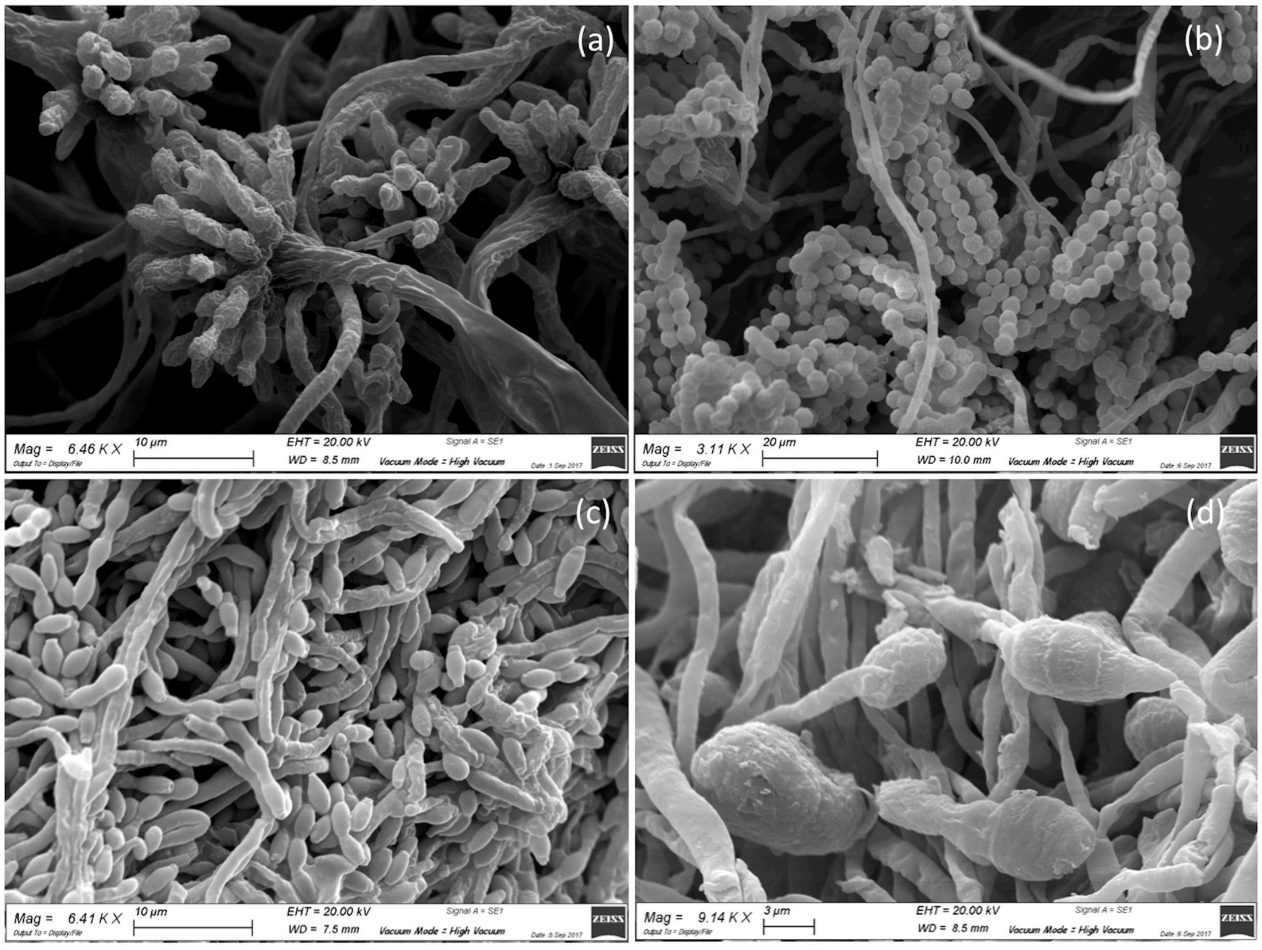
SEM analysis of molds isolated from the *recto* of the painting. Microphotographs show: a) conidial heads of *Aspergillus* spp. (magnification 6.46 KX); b) conidial heads of *Penicillium* spp. (magnification 3.11 KX); c) elliptical conidia ascribable to *Cladosporium* spp. (magnification 6.41 KX); d) club-shaped and septate spores of *Alternaria* spp. (magnification 9.14 KX).

**Fig 5:**
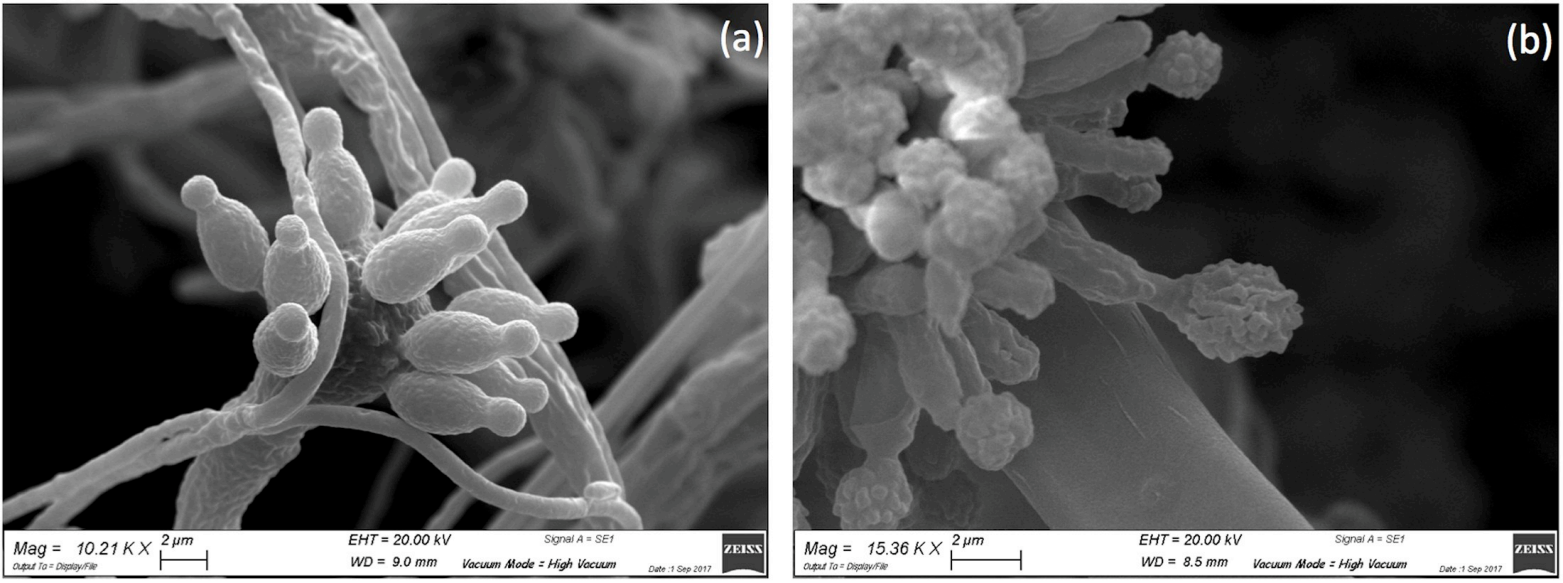
SEM photomicrographs of conidia of *Aspergillus* species with different wall ornamentations. a) conidia with a smooth surface detected on samples taken from a light-colored area (magnitude 10.21 KX), b) conidia with a slightly rough surface (typical of *A*. *ochraceus*) identified on samples collected from a dark-brown area (magnitude 15.36 KX).

Microbial isolates were initially grouped according to their colony morphology displayed on Petri dishes containing Mego agar medium. The fungi isolates were assigned to the *Aspergillus*, *Penicillium*, *Cladosporium*, and *Alternaria* genera based on the macro- and micro-morphological features of each microbial isolate determined by culture methods and OM (Fig 3) and SEM (Fig 4) observations. All of these microorganisms are frequently found on cultural heritage manufacts [56–58]. The growth of fungi on the *recto* of the painting was different in relation to that on the colors of the pictorial layer. In the samples taken from dark-brown- and red-colored areas, environmental yeasts and molds were detected; among the latter, *Aspergillus* spp. and *Penicillium* spp. were identified based on their typical morphology [59–62]. Ascomycetes such as *Aspergillus* and *Penicillium* are often found in libraries or museums on paper material and on paintings [58]. Typical conidial heads of *Aspergillus* species are shown in Figs 3a, 4a and 5, and different conidial wall ornamentations were detected in relation to the color of the area from which the sample was taken. For example, in a dark-brown area, conidia with distinctive wall ornamentations of *Aspergillus ochraceus* were visible (Fig 5b) [63,64]. Species of *Penicillium* were, instead, identified by unbranched conidiophores characterized by their typical brush-like structure (Figs 3b and 4b).

In samples taken from a lighter area, such as areas with a yellow or ruddy pink color, elliptical spores with a smooth surface ascribable to *Cladosporium* spp. were observed (Figs 3c and 4c) [65,66]. The attachment scars on *Cladosporium* conidia that form long simple chains are shown in Figs 3c and 4c. However, *Alternaria* strains were detected only in the part of the painting that was in contact with the basilica floor; it has been reported, in fact, that high values of relative humidity (more than 65%), in our case probably because of moisture capillarity, and temperature (26.8–26.9 °C) provide suitable environmental conditions for fungi growth, including *Alternaria* spp. [67]. The typical club-shaped and septate spores of *Alternaria* spp., in fact, were observed in samples taken from the lower part of the painting (Fig 3) [68], where single spores of *Alternaria* spp. with a slightly rough surface and divisions in the longitudinal and transverse directions were observed.

**Fig 6:**
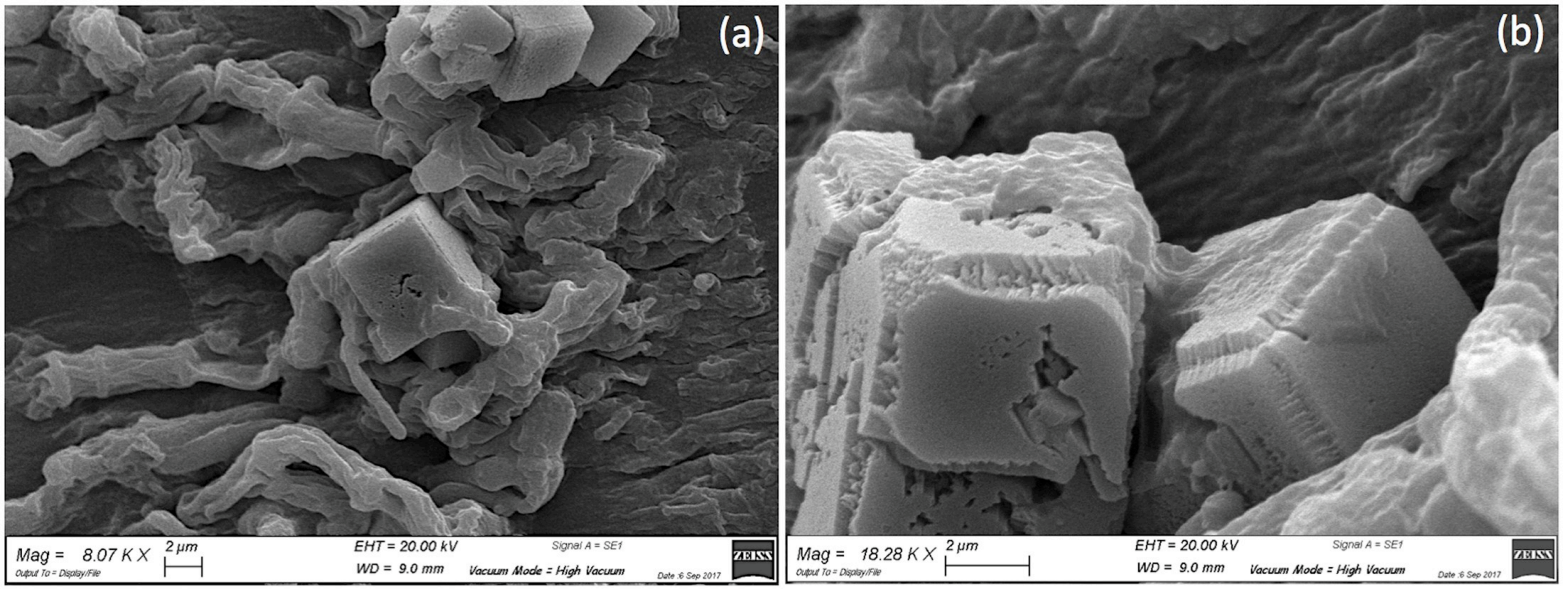
SEM photomicrographs of mycogenic minerals tangled in fungal hyphae. Original magnification a) 8.07 kx and b) 18.28 kx. The crystals were detected in samples taken from the side of the painting touching the basilica floor.

Moreover, SEM analysis of a sample collected from the same area revealed calcite crystals that were tangled in fungal hyphae (Fig 6). These crystals may be due to the ability of some species to precipitate minerals, in particular calcite and weddellite [69–72]. In this case, the precipitation of mycogenic minerals may be related to the interaction among *Penicillium* spp., *Alternaria* spp., and the preparatory layer, which is mainly composed of calcium and an organic binder. Similar results, in fact, were obtained by Unković *et al*. [73] with microfungi isolated from a deteriorated mural painting in a Serbian Church in Serbia and cultured on CaCO_3_ glucose agar plates.

**Fig 7:**
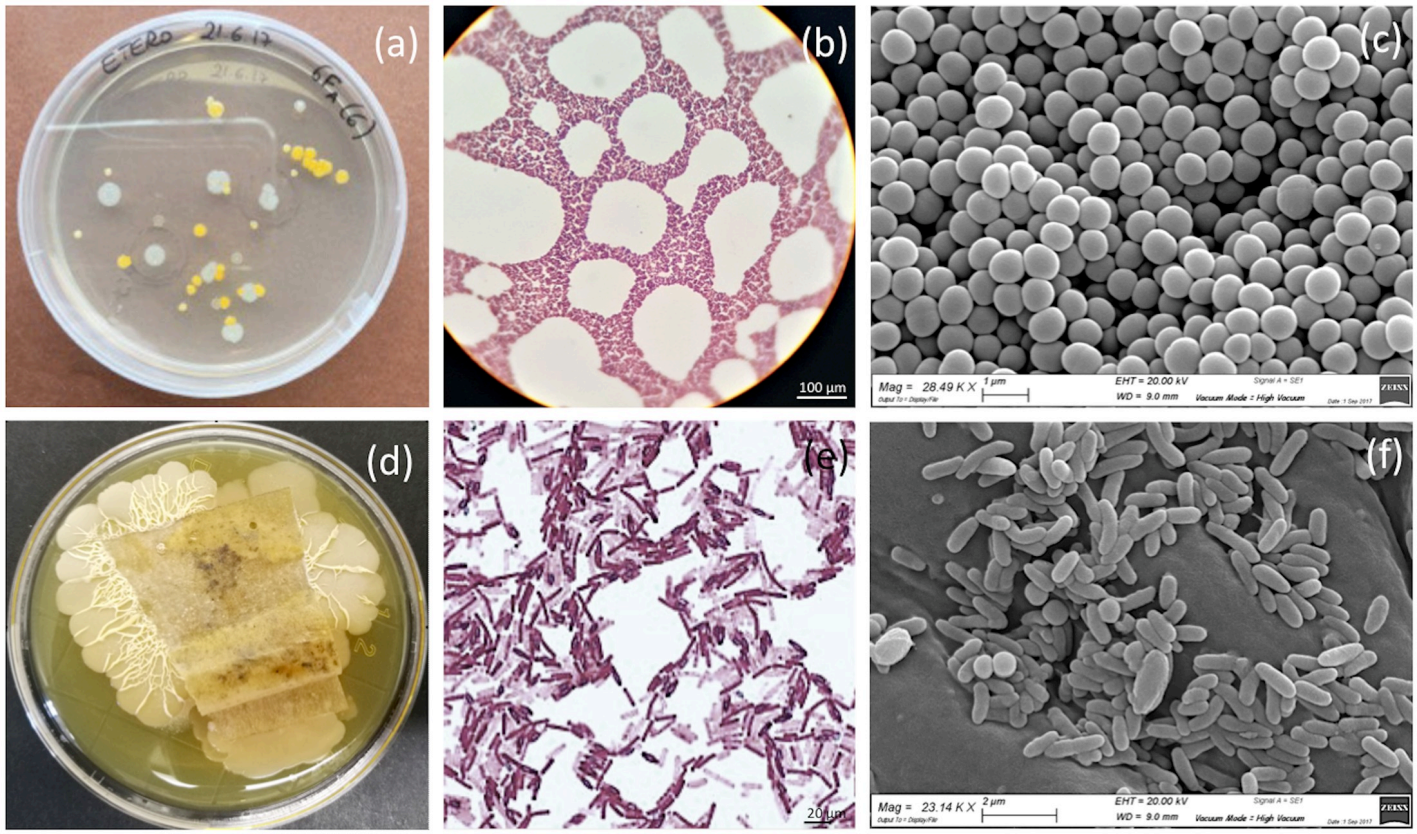
Bacteria detected on the painting. Samples were collected from the *recto* (a, b, c) and the *verso* (d, e, f) of the painting. a) colonies of *Staphylococcus* spp. on a Mego agar plate; b) the same *Staphylococcus* spp. viewed by OM after Gram staining (original magnification 100X) and c) by SEM. d) colonies of *Bacillus* spp. on a TSA agar plate; e) the same Bacillus spp. viewed by OM after Gram staining (original magnification 100X) and f) SEM.

Concerning bacteria, the microbiological study allowed the characterization of diverse bacterial strains, including gram-positive cocci and bacilli (Fig 7). Culture-dependent techniques and SEM observations revealed the presence of *Staphylococcus* spp. on the *recto* of the painting and of *Bacillus* spp. on the *verso*. In Fig 7, colonies formed by spheroid-shaped *Staphylococcus aureus* bacteria (Fig 7a, 7b and 7c) and rod-shaped bacteria ascribable to *Bacillus* spp. (Fig 7d, 7e and 7f) are shown.

Finally, the analysis did not reveal the presence of autotrophic microorganisms, probably due to low daily PAR intensity values, as detected during the monitoring campaign in the basilica (0–1.6 μmol_photons_ m^-2^ s^-1^).

### *In vitro* inhibition of microbial growth by a biocompound

**Fig 8:**
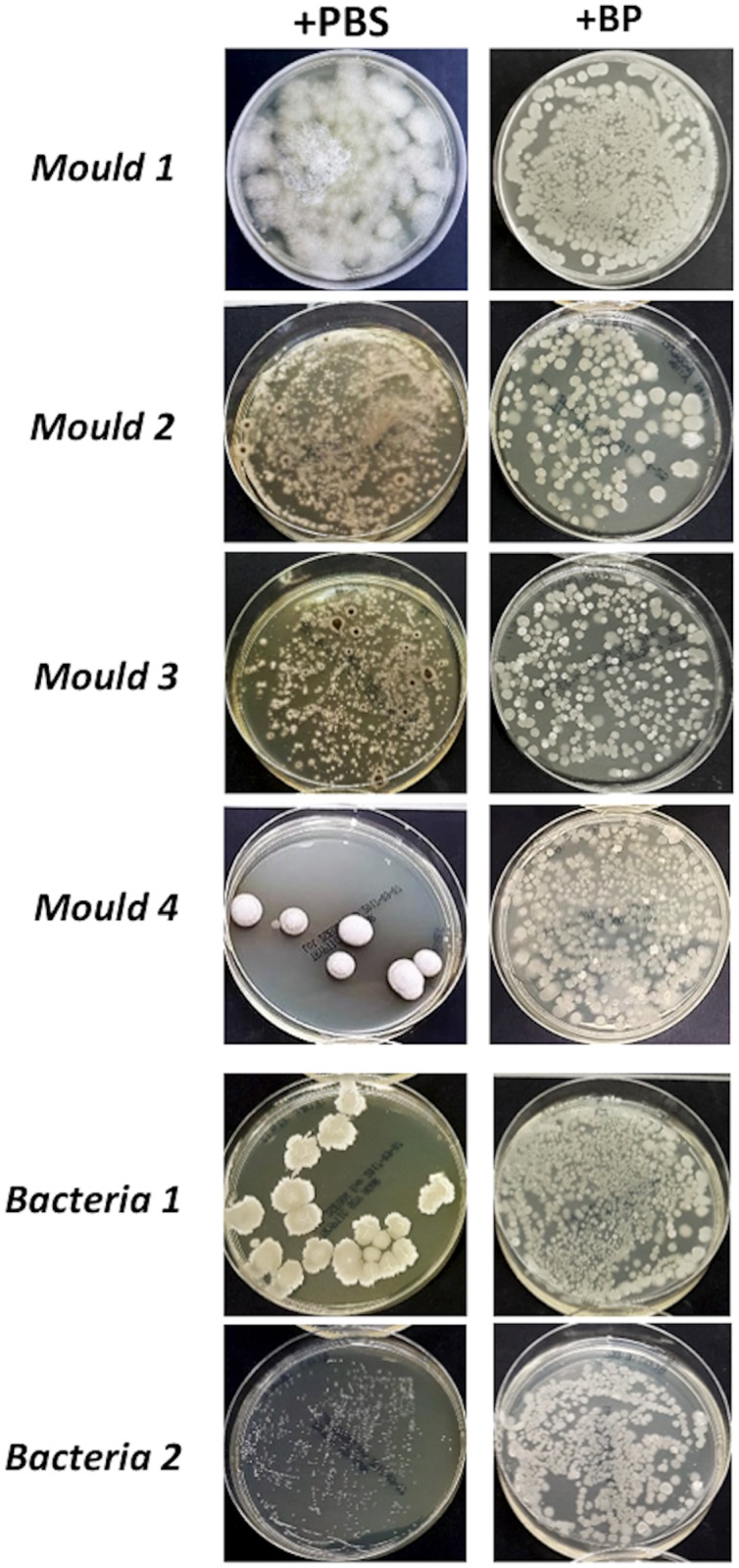
Effectiveness of a probiotic compound at inhibiting the growth of microorganisms contaminating the painting. Molds and bacteria were isolated from the painting as described in the methods. Each individual species was incubated in TSB at room temperature for 24 hours under mild agitation and then spread on TSA plates in the presence or absence of an equal amount of the probiotic product. The growth of contaminating microorganisms was evaluated after 2–5 days of incubation at room temperature. The results shown are those obtained after 5 days of incubation and are representative of three independent experiments.

To investigate the potential antimicrobial activity of the *Bacillus*-based biocompound, growth inhibition tests were performed *in vitro*. Briefly, bacteria and molds isolated from the painting were seeded on general-purpose or selective agar media in the presence or absence of the biocompound, which contained spores of *B*. *subtilis*, *B*. *pumilus*, and *B*. *megaterium*. The growth of the plated microorganisms was checked after 1, 3, and 7 days of co-culture. The results (summarized in Fig 8) indicated that the presence of *Bacillus* almost totally inhibited the development of all the tested microbes isolated from the painting, independent of their nature (mycetes or bacteria), suggesting a non-specific inhibitory mechanism mostly due to competitive antagonism, as has already been observed in other environments [28,34].

## Discussion

The present study reports the simultaneous characterization of the pictorial materials and microbial communities dwelling on the ancient oil painting “Incoronazione della Vergine” (Carlo Bononi, 17^th^ century).

The chemical compositions of the detected pigments are compatible with typical 17^th^ century paintings, and the results confirmed the *modus pingendi* of the artist [47]. The analysis, in fact, revealed the use of a brownish ground layer, mainly composed of gypsum, red ochre, and organic binder, and several natural pigments, such as natural earth (red, yellow, and brown earths), lead- based pigments (minium, biacca, lead tin yellow), cinnabar, red lac, and blue and green copper carbonate hydroxide pigments.

Some of the identified pigments, namely red lac and red and yellow earths, can be utilized as nutrient sources by painting-associated microorganisms, as previously reported in the literature [14,53–55,74]. Consistent with this, several microbes were detected on the painting, and interestingly, their presence and distribution were different in different areas of the canvas. Specifically, we observed a distinct pattern of microorganisms colonizing the painting, correlating with specific dark- or light-colored zones and suggesting specific metabolisms depending on the chemical nature of the pictorial substrate. Among the bacteria, both *Staphylococcus* and *Bacillus* genera were detected among the microorganisms dwelling on the painting. Culture-dependent techniques and SEM/EDS surveys revealed the presence of *Staphylococcus* spp. on the *recto* of the painting and of *Bacillus* spp. on the *verso*. Both of these species have been previously reported as potential contaminants of paintings and associated with biodegradation [4,12–14]. Our results confirmed that they could be found at a high frequency on oil paintings, providing evidence that Staphylococci are painting-colonizing microbes and not were just human skin contaminants transported accidentally to the canvas.

Interestingly, different bacterial genera were observed in different areas of the painting, confirming the presence of complex and different microbial ecosystems on oil paintings that strictly depending on the light, temperature, humidity, and nutrient conditions. In addition to bacterial strains, as expected, our analyses revealed the presence of filamentous fungi of the genera *Aspergillus*, *Penicillium*, *Cladosporium*, and *Alternaria*. Moreover, in this case, the distribution of the detected fungi was different in various areas of the painting. In particular, *Aspergillus* spp. and *Penicillium* spp. were detected on dark-brown (i.e., clothing of the angel) and red-colored areas (e.g., light-red cloak of the Creator), whereas *Cladosporium* spp. were observed in samples taken from lighter areas (e.g., the yellow in the sky and the ruddy pink areas). Finally, *Alternaria* spp. was detected only in the portion of the painting that was in contact with the basilica floor, where a more suitable environmental condition for its growth was probably created due to moisture capillarity. However, future works should include the use of different culture media or dilution series prior to inoculation to minimize the risk that faster-growing species might mask the growth of slower species. Moreover, future works would benefit from the use of 16S ribosomal molecular techniques to obtain a detailed description of the painting microbiota. In fact, although the detection and identification of mycetes and algae are not possible with this type of analysis, it might be extremely useful for identifying all contaminating bacterial without the limits of culture methods.

Following the isolation of the microbial species colonizing the painting, we also aimed to evaluate the potential of a biological approach to prevent their growth through competitive antagonism. In particular, we evaluated *in vitro* the potential antimicrobial activity of a bio-compound containing spores of *Bacillus* spp. *(*namely *B*. *subtilis*, *B*. *pumilus*, and *B*. *megaterium*) that were previously shown to be able to counteract the growth of many types of environmental microorganisms [28,34,35]. The results showed that *Bacillus* almost completely inhibited the growth of all microbes isolated from the painting, independent of their nature (mycetes or bacteria). These observations suggest a non-specific inhibitory action of *Bacillus*, mostly due to competitive antagonism, as already observed in other environments [28,34]. These results, although preliminary, suggest that bacteria belonging to the *Bacillus* genus might be able to also counteract *in situ* the growth of contaminating microorganisms potentially damaging to artwork and will guide future studies to assess the potential interaction of *Bacillus* with the pictorial matrix in order to identify any potential risk of direct damage to paintings caused by *Bacillus* itself. If such studies yield positive results, they could provide novel solutions for preventing biodeterioration of paintings and artwork in general.
